# In-depth characterization of trypsin-like serine peptidases in the midgut of the sugar fed *Culex quinquefasciatus*

**DOI:** 10.1186/s13071-015-0985-0

**Published:** 2015-07-16

**Authors:** André Borges-Veloso, Leonardo Saboia-Vahia, Geovane Dias-Lopes, Gilberto B. Domont, Constança Britto, Patricia Cuervo, Jose B. De Jesus

**Affiliations:** Laboratório de Biologia Molecular e Doenças Endêmicas, Instituto Oswaldo Cruz, FIOCRUZ, Rio de Janeiro, RJ Brazil; Laboratorio de Pesquisa em Leishmaniose, Instituto Oswaldo Cruz, FIOCRUZ, Av. Brasil 4365, Manguinhos, Pav. Leônidas Deane, Sala 509, CEP: 21040-360 Rio de Janeiro, RJ Brazil; Unidade de Proteômica, Laboratório de Química de Proteínas, Instituto de Química, Universidade Federal do Rio de Janeiro, Rio de Janeiro, Brazil; Departamento de Medicina, Faculdade de Medicina, Universidade Federal de São João del Rei, São João del Rei, MG Brasil

**Keywords:** *Culex quinquefasciatus*, Trypsin-like serine peptidases, Zymography, Mass spectrometry

## Abstract

**Background:**

*Culex quinquefasciatus* is a hematophagous insect from the Culicidae family that feeds on the blood of humans, dogs, birds and livestock. This species transmits a wide variety of pathogens between humans and animals. The midgut environment is the first location of pathogen-vector interactions for blood-feeding mosquitoes and the expression of specific peptidases in the early stages of feeding could influence the outcome of the infection. Trypsin-like serine peptidases belong to a multi-gene family that can be expressed in different isoforms under distinct physiological conditions. However, the confident assignment of the trypsin genes that are expressed under each condition is still a challenge due to the large number of trypsin-coding genes in the Culicidae family and most likely because they are low abundance proteins.

**Methods:**

We used zymography for the biochemical characterization of the peptidase profile of the midgut from *C. quinquefasciatus* females fed on sugar. Protein samples were also submitted to SDS-PAGE followed by liquid chromatography–tandem mass spectrometry (LC–MS/MS) analysis for peptidase identification. The peptidases sequences were analyzed with bioinformatics tools to assess their distinct features.

**Results:**

Zymography revealed that trypsin-like serine peptidases were responsible for the proteolytic activity in the midgut of females fed on sugar diet. After denaturation in SDS-PAGE, eight trypsin-like serine peptidases were identified by LC-MS/MS. These peptidases have structural features typical of invertebrate digestive trypsin peptidases but exhibited singularities at the protein sequence level such as: the presence of different amino acids at the autocatalytic motif and substrate binding regions as well as different number of disulfide bounds. Data mining revealed a group of trypsin-like serine peptidases that are specific to *C. quinquefasciatus* when compared to the culicids genomes sequenced so far.

**Conclusion:**

We demonstrated that proteomics approaches combined with bioinformatics tools and zymographic analysis can lead to the functional annotation of trypsin-like serine peptidases coding genes and aid in the understanding of the complexity of peptidase expression in mosquitoes.

## Background

The mosquito *Culex quinquefasciatus* is widespread in tropical and subtropical regions of the world, and it is adapted to urban/peri-urban areas. Despite having anthropophilic and endophilic habits, adult females exhibit high plasticity in their feeding behavior that characterizes this species as an opportunistic insect that feeds on the blood of humans, dogs, birds and livestock. This feature makes this species important in the zoonotic transmission of a wide variety of pathogens between humans and animals [1, 2]. *C. quinquefasciatus* is implicated in the dissemination of several arboviruses such as West Nile virus, St. Louis encephalitis virus, and Venezuelan equine encephalitis virus, and it has also been implicated in the transmission of protozoan parasites such as *Plasmodium relictum*. In addition, this species plays an important role as a vector of helminths such as the causative agent of lymphatic filariasis, *Wuchereria bancrofti*, and the dog heartworm, *Dirofilaria immitis* [3–8].

In mosquitoes, the main proteolytic enzymes responsible for food digestion are trypsin- and chymotrypsin-like serine peptidases as well as carboxy and amino-exopeptidases [9–12]. Trypsin-like peptidases (EC 3.4.21.4) belong to serine peptidases family S1 characterized by the His, Asp, and Ser amino acids residues within the catalytic triad [13]. It has been observed that trypsin-like serine peptidases are a multi-gene family that can be expressed as different isoforms under distinct physiological conditions [14–16]. Whereas the expression of some trypsin genes is constitutive, the expression of other trypsin genes is induced by the blood meal; hence, the expression pattern of trypsin-coding genes is biphasic [10, 16–22]. However, the confident assignment of the trypsin genes that are expressed under each condition is still a challenge due to the large number of trypsin-coding genes in the Culicidae family [7, 14, 23]. For example, 380 serine peptidase genes were reported in the genome of *Aedes aegypti* [14], but only six trypsin-like enzymes have been characterized at the protein level in the midgut tissue [12, 17, 24].

In addition to their role in food digestion, trypsin-like serine peptidases have been described as key mediators of pathogen-vector interaction. Among several midgut trypsin isoforms in *Ae. aegypti*, only one could limit Dengue virus-2 (DENV-2) infectivity [25]. Although the proteolytic environment of the midgut lumen could lead to pathogen degradation and consequently limit infectivity, arboviruses from different families such as DENV-2 (Flaviviridae), La Crosse virus (Bunyaviridae) and blue tongue virus (Reoviridae) use vector midgut peptidases for the proteolytic processing of virion surface proteins, increasing viral binding to midgut cells [26–30]. In addition, *Ae. aegypti* secreted trypsin peptidases activate a *Plasmodium gallinaceum* chitinase that is essential for peritrophic matrix evasion [31, 32]. Thus, not only the time course and the quantity of peptidase expression in the initial time of feeding does influence the infection, replication and dissemination of pathogens, but the quality of these peptidases could also be important for this interaction.

In the midgut of *C. quinquefasciatus,* trypsin-like serine peptidases have been detected after blood feeding [33]. However, the “peptidase status” of the midgut when the blood arrives into the lumen corresponds to that set by sugar feeding. Nevertheless, the expression of peptidase genes in the midgut of mosquitoes fed on sugar as well as the identity of the peptidases expressed (if any) when mosquitoes are feeding on sugar remains elusive, most likely because of the abundance of these enzymes is not enough to detect them [15, 24].

Herein, we focused on the characterization and identification of trypsin-like serine peptidases constitutively expressed in the midgut of females of *C. quinquefasciatus* that were fed only sugar. We used zymography for the biochemical characterization of the enzymes and SDS-PAGE followed by liquid chromatography–tandem mass spectrometry (LC–MS/MS) analysis for protein identification. Eight trypsin-like serine peptidases were identified by MS/MS and their molecular features were analyzed by bioinformatic tools.

## Methods

### Chemicals

All reagents were purchased from Sigma (St. Louis, MO, USA) or Merck (São Paulo, SP, Brazil). MilliQ-purified water (Millipore Corp., Bedford, MA, USA) was used to prepare all of the solutions.

### Insects

Experiments were carried out using 5-day-old *C. quinquefasciatus* female adults (Colônia strain) from a closed colony reared in the Laboratório de Fisiologia e Controle de Artrópodes Vetores - Instituto Oswaldo Cruz, FIOCRUZ, Rio de Janeiro. Larvae of *C. quinquefasciatus* were reared in plastic basins (33 × 24 × 8 cm) containing 1 L of dechlorinated water and 1 g of cat food (FriskiesW, Purina, Camaquã/RS). Larvae were kept in a biological oxygen demand incubator (BOD) at 25 ± 1 °C, with a relative humidity of 60 ± 10 % and a light:dark photoperiod of 14:10 h. The adult mosquitoes were maintained on a 10 % sucrose diet.

### Midgut dissection

The mosquitoes were anesthetized on ice and decapitated. Dissection was performed in cold PBS buffer, pH 7.4 (150 mM NaCl, 10 mM Na_2_HPO_4_). The thorax of each decapitated mosquito was immobilized with forceps (#5) and the gut, Malpighian tubules and gonads were dissected by gently pulling at the eighth abdominal segment region with another pair of forceps. The Malpighian tubules, hindgut and gonads were cut away, and the midguts were delicately washed twice with PBS buffer and transferred to a microcentrifuge tube containing the specific lysis buffers for proteome or zymography analysis. In addition, optical differential interference contrast microscopy (DIC) was used to record images from different midgut dissected samples obtained for both zymographic analysis and mass spectrometry. Such images were made with the main objective to verify the quality of the midgut dissections, *i. e*. to verify if the cuts were done consistently in the same regions of the gut and also to rule out the possibility of contaminations with metamorphosis remaining tissues into the midgut lumem.

### Zymography assays

A pool of 20 midguts were lysed with a VWR® disposable pellet mixer and cordless motor, and homogenized in a plastic eppendorff microtube containing a lysis buffer with10% glycerol, 0.6 % Triton X-100, 100 mM Tris–HCl pH 6.8 and 150 mM NaCl. The homogenate was centrifuged at 14,000 x*g* at 4 °C for 15 min, and the supernatant was collected. The protein concentration of the resulting extracts was determined using the Pierce 660 nm Protein assay (Thermo Scientific). For protein separation, 10 μg of protein were loaded in 10 % polyacrylamide gels copolymerized with 0.1 % porcine gelatin as the substrate. Electrophoresis was performed at 4 °C at a constant voltage of 110 V. Peptidase activity was detected as previously reported with few modifications [34]. The gels were incubated at 37 °C for 2, 4, 6 or 12 h in reaction buffer containing 100 mM sodium acetate (at pH 3.5 or 5.5) or 100 mM Tris–HCl (pH 7.5 or 10.0). Substrate degradation was visualized as clear bands after staining the gels with 0.2 % Coomassie blue R-250 in methanol/acetic acid (40:10) and destaining in 10 % acetic acid. The relative molecular masses of the bands were estimated by comparison with the mobility of a commercial molecular mass standard (PageRuler™ Protein Ladder, Fermentas). To determine the classes of peptidases detected by zymography, peptidase inhibition assays were conducted. Midgut homogenates were pre-incubated (before electrophoresis) for 30 min at 4 °C with one of the following peptidase inhibitors: 20 μM E-64, 1 mM phenylmethylsulfonyl fluoride (PMSF), 100 μM tosyl-L-lysyl-chloromethane hydrochloride (TLCK), 100 μM tosyl-phenylalanyl-chloromethyl ketone (TPCK), 10 μM pepstatin-A or 10 mM 1,10-phenanthroline. After electrophoresis, inhibitors were added to the reaction buffer at the same concentration, the gels were incubated during 12 h at 37 °C, and the peptidases were resolved as described above. The results were derived from three independent experiments carried out in triplicate.

### In vitro enzyme assays

The effects of pH and peptidase inhibitors on the proteolytic activities of midgut homogenates were also evaluated *in vitro* using the fluorogenic substrate 7-amido-4-methylcoumarin hydrochloride (Z-Phe-Arg-AMC). For each assay, 100 μM of substrate were used. The reactions were initiated as described previously [34]. Briefly, 10 μg of protein from the midgut were diluted in 100 mM sodium acetate (at pH 3.5 or 5.5), 100 mM Tris–HCl (pH 7.5 or 10.0) with or without inhibitor addition. The fluorescence intensity was evaluated by spectrophotofluorometry for 60 min (SpectraMax Gemini XPS, Molecular Devices, CA) using excitation and emission wavelengths of 380 and 460 nm, respectively. As a blank, the substrate (100 μM) was diluted in a reaction buffer containing 100 mM sodium acetate (at pH 3.5 or 5.5) or 100 mM Tris–HCl (pH 7.5 or 10.0). The value of the blank was automatically subtracted by the fluorometer software (SoftMax®Pro, Molecular Devices, CA) when the data were acquired. All assays were performed at 37 °C. The results were derived from three independent experiments performed in triplicate.

### Sodium dodecyl sulfate polyacrylamide gel electrophoresis (SDS-PAGE), protein digestion and peptide extraction

Fifty pooled midguts were directly lysed in Laemmli sample buffer containing 4 % SDS, 20 % glycerol, 10 % 2-mercaptoethanol, 0.004 % bromophenol blue and 0.125 M Tris-HCl, pH approx. 6.8. Lysis was performed by mechanical homogenization using a plastic pestle. The lysate was centrifuged twice at 14,000 x*g* for 10 min at 4 °C and the proteins in the resulting supernatant were collected. The protein concentration was determined using the Pierce 660 nm Protein assay (Thermo Scientific). Then, the samples were heated for 5 min in a boiling water bath and separated by 12 % SDS-PAGE, 30 % acrylamide, 0.8 % bis-acrylamide. Proteins were stained using Coomassie Brilliant Blue and photo-documented. Three gels from three independent midgut suspensions were performed. Proteins were enzymatically digested following procedures previously described [35] with some modifications. Briefly, fine slices from each protein lane were manually excised and de-stained three times in 400 μL of 50 % acetonitrile, 25 mM NH_4_HCO_3_ pH 8.0 for 15 min. Proteins were subsequently reduced and alkylated using 65 mM dithiothreitol (DTT) and 200 mM iodoacetamide, respectively. Gel slices were washed with 100 mM NH_4_HCO_3_ followed by dehydration with acetonitrile. Slices were rehydrated with a solution of 20 ng/μL of sequencing grade modified porcine trypsin (Promega, USA) in 50 mM NH_4_HCO_3_ and incubated overnight at 37 °C. Peptides were extracted using 0.1 % formic acid in 50 % v/v acetonitrile, desalted and concentrated with Poros oligo R3 C18 resin (Applied Biosystems, USA). The eluted peptides were loaded in a nano-high performance liquid chromatography (nanoHPLC) in-line with a hy'brid linear trap quadrupole (LTQ) Orbitrap mass spectrometer.

### Mass spectrometry analysis

For each sample 4 μL of peptides solution (0.1 % formic acid) were applied to an EASY II-nanoHPLC system (Thermo Fisher Scientific) coupled online to an electrospray (ESI)-LTQ-Orbitrap Velos mass spectrometer (Thermo Fisher Scientific). Peptides were eluted through a trap column (150 μm × 2 cm) packed in-house with C-18 ReproSil 5 μm resin (Dr. Maisch) and an analytical column (100 μm x 15 cm) packed in-house with C-18 ReproSil 3 μm resin (Dr. Maisch) using a mobile phase A of 0.1 % (v/v) formic acid in water and a mobile phase B 0.1 % (v/v) formic acid in acetonitrile. Gradient conditions were as follows: 5 to 40 % B in 180 min. Mass spectra were acquired in the positive mode using a data-dependent automatic (DDA) survey MS scan and tandem mass spectra (MS/MS) acquisition. Each DDA consisted of a survey scan in a 300 − 2000 m/z range and resolution 60000 with a target value of 1 × 10^−6^ ions. Each survey scan was followed by the MS/MS of the 10 most intense ions in the LTQ using collision-induced dissociation (CID). Ions previously fragmented were dynamically excluded for 60 s.

### Database searching

Mass spectra were searched against a customized non-redundant database including sequences of all Culicidae species available at UniRef100 (101,993 sequences, downloaded May 2015, http://uniprot.org) using the Mascot MS/MS ion search engine (Matrix Science, Oxford, UK, version 2.4.1). The search parameters in the Mascot server were as follows: lack of taxonomic restrictions; one tryptic missed cleavage; carbamidomethylation of cysteine residues as a fixed modification and oxidation of methionine and acetylation as variable modifications; 10 ppm mass tolerance for the MS mode and 0.5 Da tolerance for its corresponding MS/MS fragments. Scaffold (version 4.3.0, Proteome Software Inc., Portland) was used to validate MS/MS peptide and protein identifications. Peptide identifications were accepted at 95.0 % probability by the Peptide Prophet algorithm [36] using the Scaffold delta mass correction. Protein identifications were accepted at 95.0 % probability and if they were supported by two or more independent pieces of evidence (e.g., identification of a peptide with different charge states, a modified and a non-modified version of the same peptide, or two different peptides). Protein probabilities were assigned by the Protein Prophet algorithm [37].

To confirm peptidase identifications, mass spectra were also analyzed using the ProLuCID 1.3 engine at the PatternLab platform [38] against the same customized database. Searches were performed with one missed cleavage, with carbamidomethylation of cysteine residues as a fixed modification, methionine oxidation as a variable modification and mass tolerances of 40 ppm and 0.5 Da for precursor and fragment ions, respectively. The validity of the peptide sequence matches (PSMs) was assessed using the Search Engine Processor (SEPro) at the PatternLab platform [39].

### Multiple sequence alignment and bioinformatics analysis

The complete amino acid sequences of the peptidases identified by mass spectrometry were fully retrieved from the VectorBase database (http://biomart.vectorbase.org) [40]. Multiple sequence alignments were performed using CLUSTAL Omega [41]. FASTA sequences of all trypsin identified by mass spectrometry were compared against well annotated sequences of bovine chymotrypsinogen (CTRA_BOVIN), bovine trypsinogen (TRY1_BOVIN), *Ae. aegypti* trypsin 3A1 (TRY3_AEDAE) and *An. gambie* trypsin-6 (TRY6_ANOGA). The amino acid sequence of each identified trypsin was scanned for various domains and motifs. The residues at the active site (His, Asp, Ser), the signal peptide, the conserved cysteine residues of disulfide bounds and the protein size of precursor and mature forms of peptidases were detected using the PROSCAN function of the PROSITE suite (http:// prosite.expasy.org) [42]. The signal peptide was also predicted by SignalP 4.0 (http://cbs.dtu.dk/services/SignalP) [43]. To predict N-glycosylation and O-glycosylation sites, amino acid sequences were analyzed using the NetNGlyc 1.0 Server (http://cbs.dtu.dk/services/NetNGlyc) [44] and NetOGlyc 4.0 Server (http://cbs.dtu.dk/ services/NetOGlyc) [45], respectively. To identify species-specific trypsin we used the Skyline software (http:// proteome.gs.washington.edu/software/skyline) [46] to search against the same database of Culicidae sequences used for proteomic analysis.

## Results and discussion

**Fig. 1:**
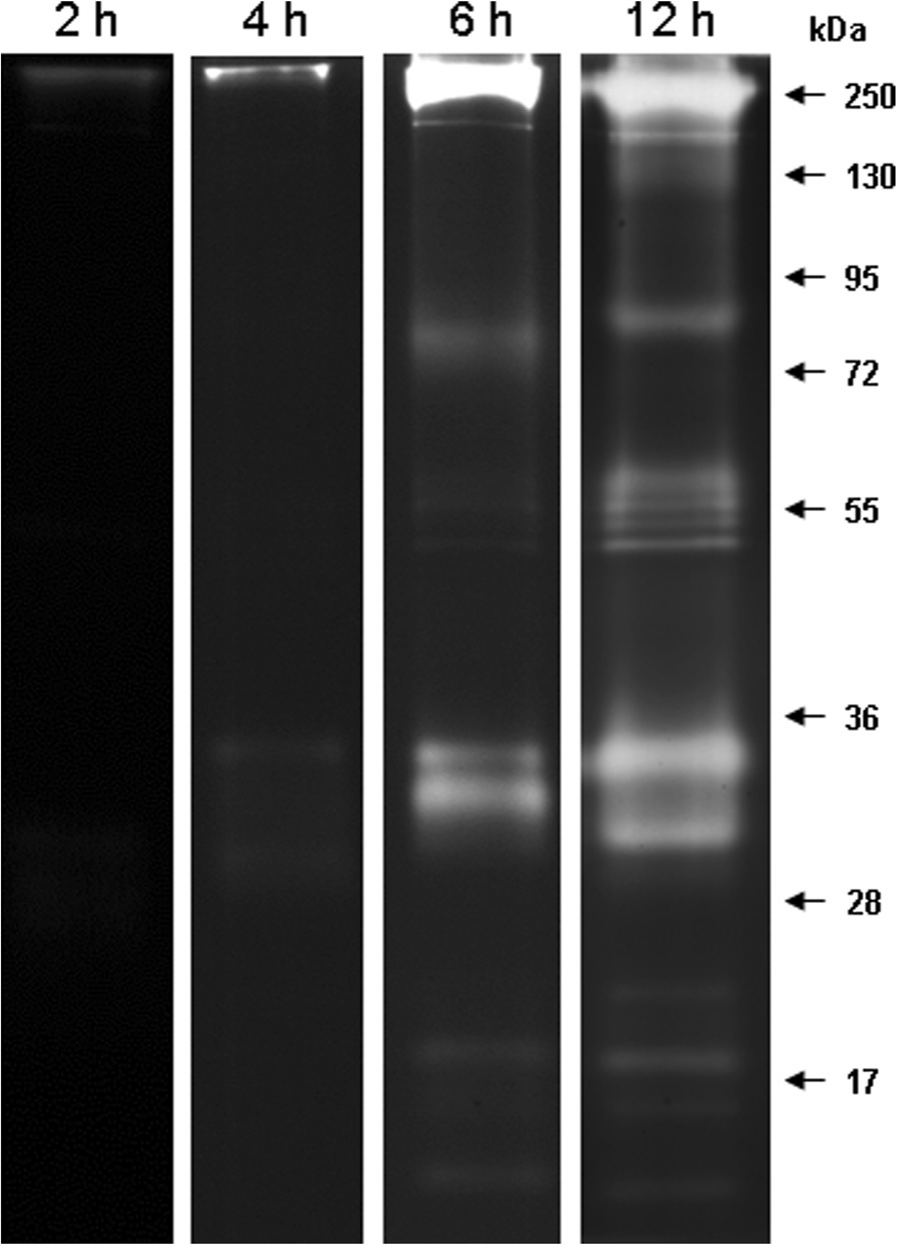
Time course of proteolytic activities exhibited by midgut extracts of female *C. quinquefasciatus* fed on sugar. Proteolytic activities were evaluated after 2, 4, 6 and 12 h incubations in 0.1 M Tris–HCl buffer (pH 7.5). The numbers on the right indicate the molecular mass of standards utilized in the gel (kDa)

Zymographic assays revealed a complex serine peptidase profile in the midgut of *C. quinquefasciatus* females composed of at least eleven bands of proteolytic activity (Fig. 1). Among these bands, 3 migrated at 28 to36 kDa, which is the expected molecular mass for monomeric trypsin [12, 17, 20, 47]. In addition, trypsin activities at high molecular mass regions such as 55, 80 and 130 kDa as well as at low molecular mass regions below 20 kDa were observed (Fig. 1). These activities could be due to (i) sample preparation, *i.e*., protein samples are not boiled in the presence of SDS and β-mercaptoethanol, therefore peptidases are not completely denatured or reduced, enabling protein aggregation and/or oligomerization that slows the electrophoretic migration; and (ii) interaction of peptidases with the substrate could also account for the slow migration [48, 49]. Despite such factors that could impede the regular migration of the peptidases, we cannot rule out the possibility that some peptidases could be extensively processed at the post-translational level, increasing their apparent molecular mass in the gel and allowing their association with other proteins in the midgut extract [49–51]. Despite these possibilities, zymographic analysis is a highly reproducible method for the study of the proteolytic profiles in different Culicidae species, suggesting that such high molecular mass enzymes are common findings and that they are not experimental artifacts [34, 52, 53]. Similarly, other authors have observed such results in other insects [54].

To further characterize the profile of proteolytic activities in the midgut of *C. quinquefasciatus* females, we performed a time-course analysis of peptidase activities over a range of 2-12 h. Although proteolytic bands began to be visualized from 6 h of incubation, the complete profile was detected after 12 h (Fig. 1). These results differ from the proteolytic activities in larval stages of *C. quinquefasciatus* [34] where activities we detected at 2 h of incubation. Such difference may be due to the fact that the larval midgut exhibits high peptidase activities that are more easily detected due to the high and constant feeding activity of larvae. Conversely, there is little peptidase activity in midgut of a sugar fed *Culex* adult female, possibly because the insect does not need it. Such results indicate that different life stages of *C. quinquefasciatus* exhibit stage-specific proteolytic profiles, which may be related to qualitative and quantitative differential expression of peptidases according to the feeding behavior.

**Fig. 2:**
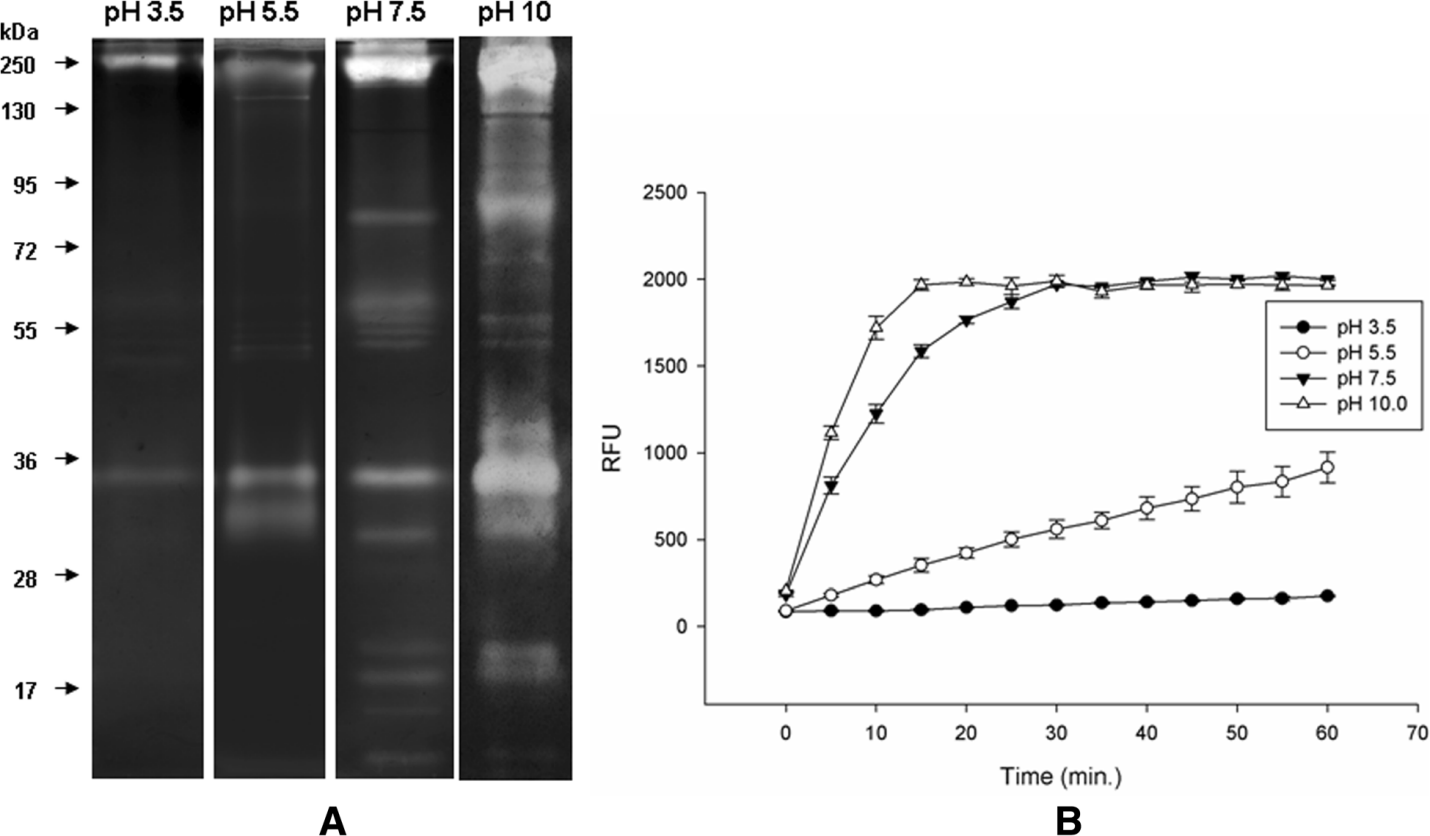
Effect of pH on the proteolytic activities of midgut extracts from *C. quinquefasciatus* females fed on sugar. **a**. The pH influence was evaluated by incubation of protein extracts at 37 °C for 12 h in 0.1 M sodium acetate buffer pH 3.5, 5.5 or 0.1 M Tris–HCl buffer pH 7.5, 10.0. The numbers on the left indicate the molecular mass of standards utilized in the gel (kDa). **b**. In-solution assays were performed using the fluorogenic substrate Z-Phe-Arg-AMC in 0.1 M sodium acetate buffer pH 3.5, 5.5 or 0.1 MTris–HCl buffer pH 7.5 or 10.0

**Fig. 3:**
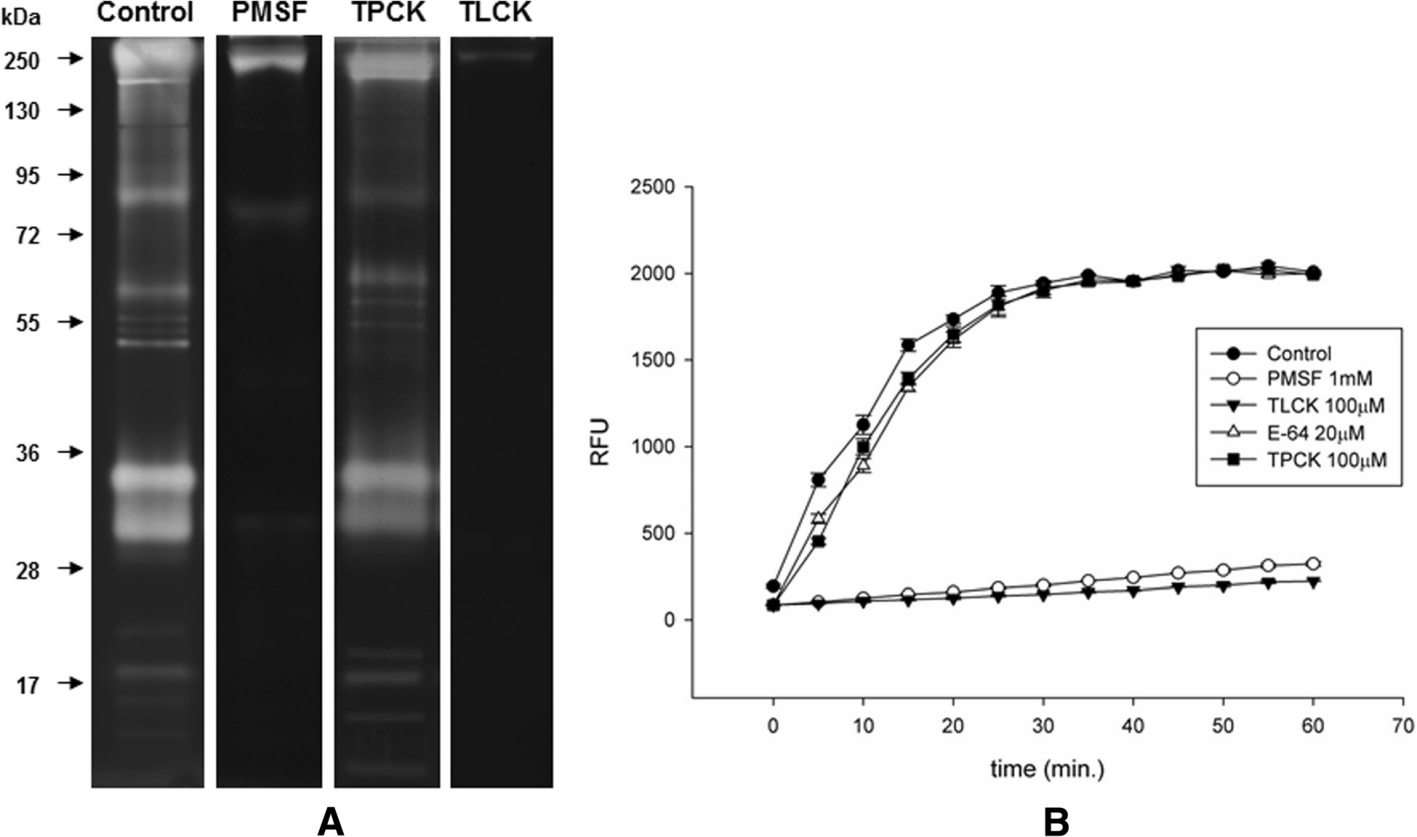
Effect of peptidase inhibitors on the proteolytic profiles of midgut extracts from *C. quinquefasciatus* females fed on sugar. **a**. Samples were pre-incubated for 30 min in the presence of 1 mM PMSF, 100 μM TLCK and 100 μM TPCK. The proteolytic activities were detected after incubating the gels for 12 h at 37 °C in Tris–HCl buffer (pH 7.5). The control was processed under the same conditions but in the absence of inhibitors. The numbers on the left indicate the molecular mass of standards utilized in the gel (kDa). **b**. The in-solution assays were performed using the fluorogenic substrate Z-Phe-Arg-AMC in 100 mM Tris-HCl buffer, pH 7.5, in the absence (control) or presence of 1 mM PMSF, 100 μM TLCK, 20 μM E-64 or 100 μM TPCK

The proteolytic activities were evaluated for pH dependence and sensitivity to inhibitors. Although weak peptidase activities are observed at acid pH, the activities increased at alkaline pH between 7.5 and 10.0 (Fig. 2). We observed high proteolytic activities at pH 10, but several bands overlapped, which impeded an accurate analysis of the proteolytic profile by zymography (Fig. 2a). For this reason, all subsequent assays were conducted at pH 7.5. Although at pH 10 we could hardly detect bands different from those observed at pH 7.5, we cannot rule out the possibility that other peptidase activities could be present at pH 10. In addition, the effect of pH on peptidase activities was also analyzed using a fluorogenic substrate (Fig. 2b). This assay corroborated the results observed by zymography and allowed a quantitative analysis of proteolytic activities at the distinct pH. In agreement with the results obtained previously for other Diptera, trypsin-like serine peptidases of *C. quinquefasciatus* are highly active at alkaline pH [34, 52, 53, 55]. PMSF, a specific inhibitor of serine peptidases, revealed that the profile of active peptidases expressed in the midgut of females fed a sugar diet is due to serine peptidases (Fig. 3). To determine whether such activities are specifically due to trypsin- or chymotrypsin-like serine peptidases, specific inhibitors TLCK and TPCK, respectively, were used. All activity bands were strongly inhibited by TLCK, indicating that the serine peptidases detected here belong to the trypsin-like family. In addition, *in vitro* assays confirmed the results obtained in the zymographic analysis. Proteolytic activities were inhibited by PMSF and TLCK but not by E-64or TPCK, inhibitors of cysteine peptidases or chymotrypsin-like serine peptidases, respectively. That means that the proteolytic profile detected under the conditions here analyzed is due to trypsin-like serine peptidases. These results agree with previous reports on the expression of trypsin-like serine peptidases in the midgut of other Culicidae [12, 14, 17, 24].

**Fig. 4:**
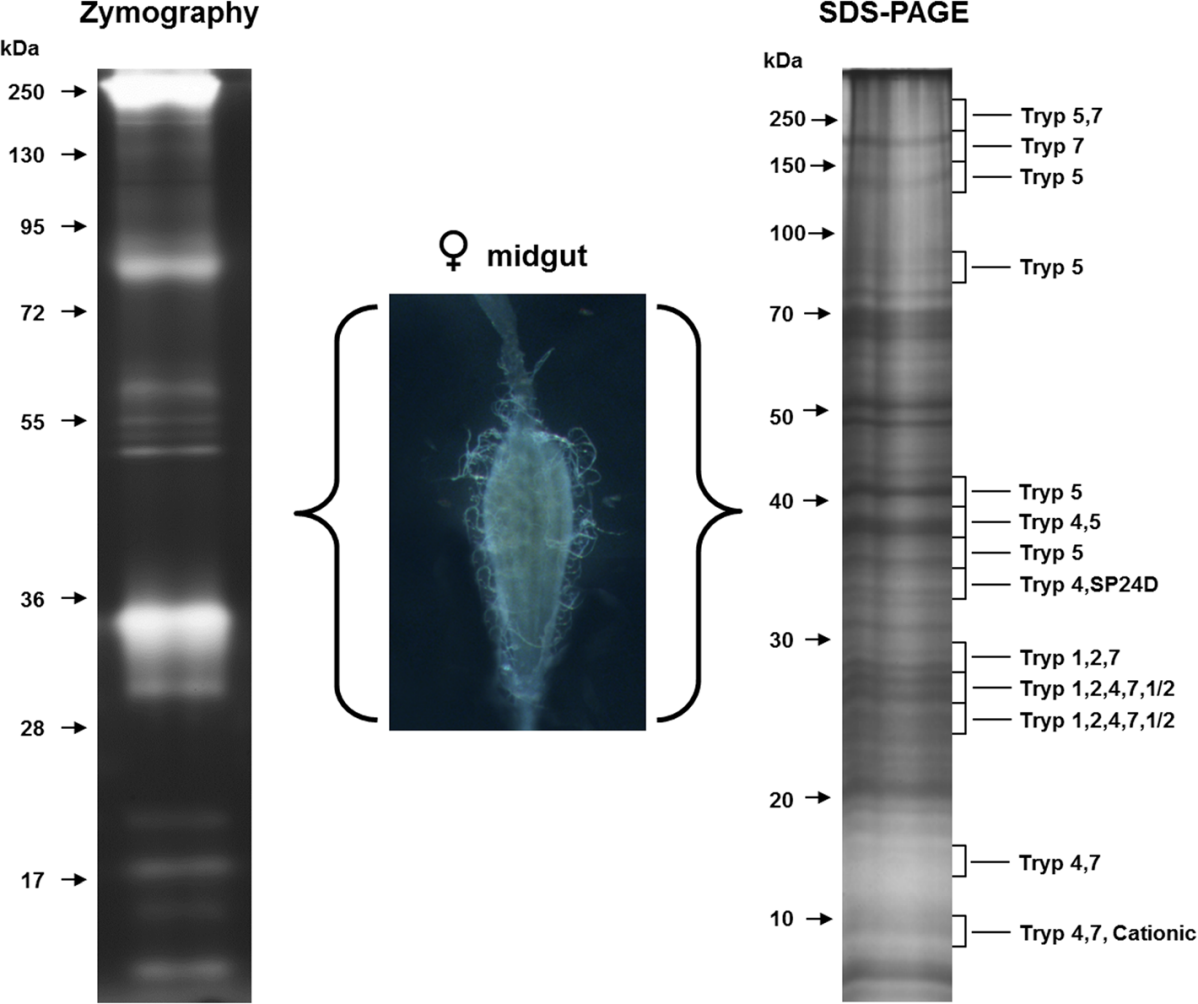
Representative zymographic profile and SDS-PAGE of total protein extracts of *C. quinquefasciatus* midgut extract. This figure shows two different electrophoretic systems used for characterize and identify, respectively, the trypsin-like serine peptidases: the zymography where proteins are resolved under non-reducing conditions and therefore their activity can be detected, and the denaturating SDS-PAGE ran under reducing conditions. SDS-PAGE slices were used for peptidase identification by mass spectrometry. The numbers on the left of each electrophoresis indicate the molecular mass of standards utilized in the gel (kDa). This figure also shows a representative image of midgut recorded by optical differential interference contrast microscopy (DIC)

**Fig. 5:**
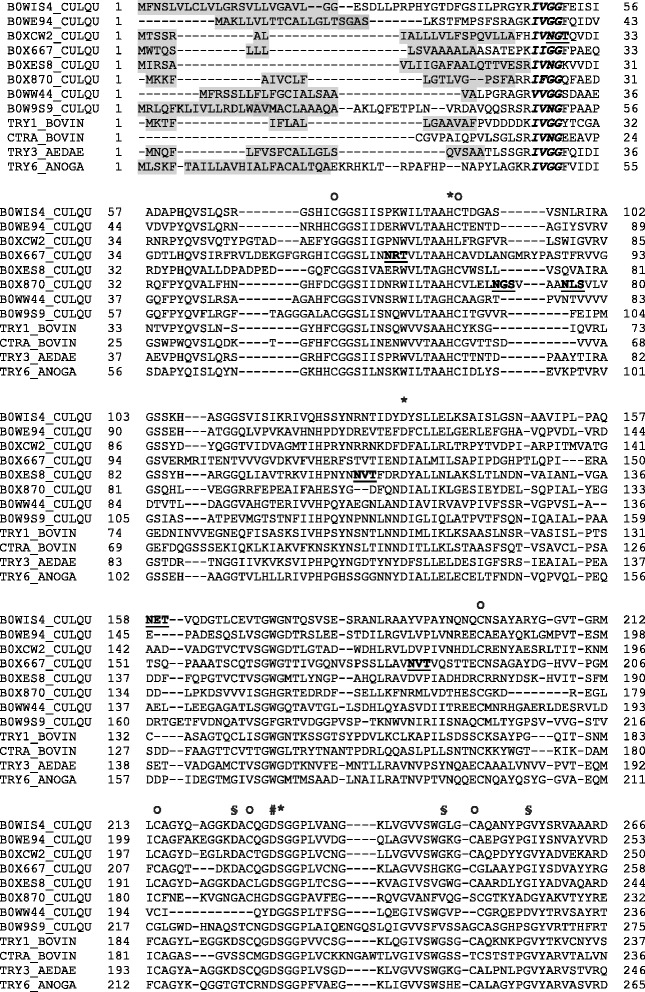
Alignment of *C. quinquefasciatus* trypsin sequences identified by MS/MS and well annotated trypsin and chymotrypsin sequences (bovine, *Ae. aegypti, An. gambiae*). Regions of importance are represented as follows: (Gray) signal peptide; (Italic and bold) N-terminal residues of the active enzyme; (O) conserved cysteine of disulfide bonds; (*) conserved catalytic triad; (§) accessory catalytic residues; (#) highly conserved Asp 194 based on bovine α-chymotrypsinogen; (underline and bold) glycosylation sites

The *C. quinquefasciatus* genome codes for 403 putative trypsin-like serine peptidase genes [14], but it is unknown which of them are expressed in the midgut tissue. Here, we identified seven trypsin-like serine peptidases constitutively expressed in the midgut of females fed a sugar diet using two independent search engines: Mascot (followed by Scaffold validation) and ProLuCID (Table 1). In addition, one trypsin-like serine peptidase was exclusively identified by MASCOT (followed by Scaffold validation) based on one peptide and one spectrum evidence (Table 1, B0WW44, gray filled). Interestingly, the SDS-PAGE bands where peptidases were identified by MS/MS coincide with the zymographic regions where peptidase activities were observed (Fig. 4). Although most of the peptidases were identified in electrophoretic bands migrating between 25 to 40 kDa (Fig. 4), the Trypsin5 and Trypsin7 were the only ones found in the high molecular mass region (Fig. 4). Conspicuously, these enzymes exhibited predicted sites for N-Glycosylation. Particularly, Trypsin5 also present predicted O-Glycosylation sites and transmembrane regions (Table 3). Such features could in fact alter the migration pattern of the mature protein. Nevertheless, as sample preparation for each electrophoresis is different, a comparison of peptidase mobility is difficult, but, in any case, both methodologies serve for mapping the identified peptidases. All identified trypsin proteins matched with *C. quinquefasciatus* protein sequences. The alignment of the full sequence of the peptidases identified by mass spectrometry showed several structural features typical of invertebrate digestive trypsin peptidases: (i) the conserved histidine, aspartic acid and serine residues forming the catalytic triad; (ii) six cysteine residues at conserved positions involved in the forming of disulfide bonds; (iii) the signal peptide sequence; (iv) the putative autocatalytic activation motifs immediately after an arginine or lysine residue (R/K- IVGG); (v) the motifs characteristic of active peptidases LTHAAC, DIAL, and GDSGGP (Fig. 5, Table 2) [56]. Interestingly, some trypsin peptidases identified here have distinct features. For example, we observed that the autocatalytic motif of Trypsin 4 has a His residue instead of R/K residues, which could suggest that this enzyme has a specific signal for activation. In addition, the activation motifs in Trypsin 5, IIGG, and cationic trypsin, VVGG, differ by one amino acid residue from the classical motif sequence (IVGG) [57, 58].

**Table 1:** Trypsin-like serine peptidases identified by mass spectrometry in the midgut of *Culex quinquefasciatus* females fed on sugar

Identified Proteins	Accession Number	Molecular Weight	Mascot exclusive peptides	Mascot total spectra	Mascot Coverage %	Peptide sequence identified by MASCOT	Mascot Ion score	Peptide sequences identified by ProLuCID	ProLuCID unique peptides	ProLuCID total spectra	ProLuCID Coverage %
Trypsin 4 OS = Culex quinquefasciatus GN = CpipJ_CPIJ017414	B0XCW2_CULQU	28 kDa	3	13	18				6	29	28
						(R)VGSSYDYQGGTVIDVAGMTIHPR(Y)	35.21	(R)VGSSYDYQGGTVIDVAGMTIHPR(Y)			
						(K)DFDFALLR(L)	52.94	(K)DFDFALLR(L)			
						(K)GCAQPDYYGVYADVEK(A)	39.12	(K)GCAQPDYYGVYADVEK(A)			
								(K)NMLCAGYDEGLR(D)			
								(R)LSWIGVR(V)			
								(R)ENYAESR(L)			
Trypsin 7 OS = Culex quinquefasciatus GN = CpipJ_CPIJ017964	B0XES8_CULQU	27 kDa	4	14	18				5	25	18
						(R)GGQLIAVTR(K)	53.31	(R)GGQLIAVTR(K)			
						(R)DYALLNLAK(S)	50.34	(R)DYALLNLAK(S)			
						(R)AVDVPIADHDR(C)	24.69	(R)AVDVPIADHDR(C)			
						(K)DACLGDSGGPLTCSGK(V)	49.46	(K)DACLGDSGGPLTCSGK(V)			
								(F)M*LCAGYDAGGK(D)			
Trypsin-5 OS = Culex quinquefasciatus GN = CpipJ_CPIJ015103	B0X667_CULQU	30 kDa	3	7	16				3	14	16
						(K)IIGGFPAEQGDTLHQVSIR(F)	35.64	(K)IIGGFPAEQGDTLHQVSIR(F)			
						(K)GCGLAAYPGIYSDVAYYR(G)	29.61	(K)GCGLAAYPGIYSDVAYYR(G)			
						(R)GWIDSCLAGK(C)	31.7	(R)GWIDSCLAGK(C)			
Trypsin-1 OS = Culex quinquefasciatus GN = CpipJ_CPIJ007079	B0WIS4_CULQU	29 kDa	3	6	18				6	10	34
						(R)IVGGFEISIADAPHQVSLQSR(G)	51.51	(R)IVGGFEISIADAPHQVSLQSR(G)			
						(K)HASGGSVISIK(R)	26.21	(K)HASGGSVISIK(R)			
						(R)AAYVPAYNQNQCNSAYAR(Y)	29.15	(R)AAYVPAYNQNQCNSAYAR(Y)			
						(K)DACQGDSGGPLVADGK(L)	33.42	(R)NTIDYDYSLLELK(S)			
								(R)GSHICGGSIISPK(W)			
								(K)WILTAAHCTDGASVSNLR(I)			
Trypsin 2 OS = Culex quinquefasciatus GN = CpipJ_CPIJ005273	B0WE94_CULQU	28 kDa	2	2	12				5	13	30
						(R)LEFGHAVQPVDLVR(D)	19.14	(R)LEFGHAVQPVDLVR(D)			
						(R)DEPADESQSLVSGWGDTR(S)	27.7	(R)DEPADESQSLVSGWGDTR(S)			
								(R)WVLTAAHCTENTDAGIYSVR(V)			
								(R)GVLVPLVNR(E)			
								(K)LGMPVTESMICAGFAK(E)			
Serine protease1/2 OS = Culex quinquefasciatus GN = CpipJ_CPIJ003826 PE = 3 SV = 1	B0W9S9_CULQU	30 kDa	2	3	18				2	3	10
						(R)TGETFVDNQATVSGFGR(T)	35.91	(R)TGETFVDNQATVSGFGR(T)			
						(R)TVDGGPVSPTK(N)	35.12	(R)TVDGGPVSPTK(N)			
Serine protease SP24D OS = Culex quinquefasciatus GN = CpipJ_CPIJ015368	B0X870_CULQU	27 kDa	1	1	10				1	3	10
						(K)LGESIEYDELSQPIALYEGDDLPK(D)	34.98	(K)LGESIEYDELSQPIALYEGDDLPK(D)			
Cationic trypsin OS = Culex quinquefasciatus GN = CpipJ_CPIJ011378	B0WW44_CULQU	26 kDa	1	1	8						
						(R)IVVHPQYAEGNLANDIAVIR(V)	32.92

**Table 2:** General characteristics of *Culex quinquefasciatus* trypsin-like serine peptidases identified by mass spectrometry ∆

Uniprot accession number	Protein name	Active sitetriad position	Cysteine pair residues	Residues confering substrate specificity	Protein size (aa)		Activation site	Conserved regions		
					Precursor	Mature		LTAAHC	DIAL	GDSGGP
B0WIS4	Trypsin 1	His88, Asp133, Ser229	73-89, 198-214, 225-249	Asp223, Gly246,Gly256	274	226	YR^IVGG	LTAAHC	DYSL	GDSGGP
B0WE94	Trypsin 2	His75, Asp120, Ser216	60-76, 183-200, 212-236	Asp210, Gly234,Gly244	261	226	GK^IVGG	LTAAHC	DFCL	GDSGGP
B0XCW2	Trypsin 4	His70, Asp116, Ser213	151-219, 181-198, 209-233	Asp207, Gly230,Gly240	258	233	FH^IVNG	LTAAHL	DFAL	GDSGGP
B0X667	Trypsin 5	His73, Asp127, Ser221	58-74, 160-227, 192-208, 217-241	Asp215, Gly238,Gly248	293	268	PK^IIGG	LTAAHC	DIAL	GDSGGP
B0XES8	Trypsin 7	His67, Asp112, Ser207	52-68, 146-213, 176-192, 203-227	Asp201, Gly224,Gly234	252	229	SR^IVNG	LTAGHC	DYAL	GDSGGP
B0X870	SP24D	His63, Asp109, Ser195	48-64, 172-181, 191-216	?	240	217	RR^IFGG	LTAAHC	DIAL	GDSGGP
B0W9S9	Serine protease 1/2	His92, Asp135, Ser233	77-93, 202-217, 229-259	?	283	235	SR^IVNG	LTAAHC	DIGL	GDSGGP
B0WW44	Cationic trypsin	His69, Asp114, Ser202	54-70	?	244	216	GR^VVGG	LTAGHC	DIAV	YDGGSP

∆ Extracted after CLUSTAL Omega aligment analysis

(aa) total number of amino acid residues

? = other residues different than DGG

^ = Indicates the clivage site for zymogen activation

An important difference between vertebrate and invertebrate trypsin is the number and location of disulfide bonds. Vertebrate trypsins commonly have six disulfide bonds, whereas, in general, trypsins from insects and crustaceans have only three disulfide bonds at conserved positions, close to the active site [56, 59]. The alignment of trypsin peptidases identified here shows that five of them, Trypsin1, Trypsin2, Trypsin4, Serine protease SP24D and Serine protease ½ have three disulfide bonds while the Trypsin5 and Trypsin7 have four disulfide bonds and the Cationic trypsin only has one. Although the number of disulfide bonds is different in the trypsins identified here, the role of the disulfide bonds is crucial for the tridimensional structure of the enzymes and, consequently, for their activity [56, 60].

Trypsin-like serine peptidases identified here were further analyzed regarding their predicted cellular location, presence of transmembrane helices, and glycosylation motifs using bioinformatics tools (Table 3). Analysis of the prediction of cellular location using the Target P server indicates that all trypsin peptidases are secreted enzymes, which is a typical feature of the digestive enzymes found in the midgut lumen [14, 15, 17]. However, the prediction of transmembrane helices, using the TMHMM server, revealed that Trypsin4 and Trypsin5 have one transmembrane domain (Table 3) suggesting that these enzymes could be targeted to the midgut membrane [55, 61]. Although glycosylation is not a common post-translational modification in trypsin, some glycosylation motifs have been observed in invertebrate trypsin [15]. Five of the eight trypsin isoforms identified here have predicted sites for O- and N-glycosylation (Table 3, Fig. 5). In agreement with this observation, trypsins peptidases from *An. gambiae* could be glycosylated and that such modification might be required for the association of peptidases with peritrophins in the peritrophic membrane [51].

**Table 3:** In silico characterization of trypsin-like serine peptidases identified in the midgut of *Culex quinquefasciatus* females fed on sugar

Uniprot accession number	Protein name	Target P prediction ①	Signal P prediction ②	TMHMM prediction ③	N-Glycosylation prediction ④	O-Glycosylation prediction ⑤	Exon number ⑥	Paralogues number ⑥	Supercontig ⑥
B0WIS4	Trypsin 1	S (0.901)	23^24 (0.761)	No	158-NETV (0.7243)	36-T (0.627995)/40-S (0.689668)	1	36	3.14
B0WE94	Trypsin 2	S (0.910)	18^19 (0.818)	No	No	No	2	36	3.94
B0XCW2	Trypsin 4	S (0.973)	22^23 (0.935)	inside: 1-6/Tmhelix:7-26/outside: 27-258	27-NGTQ (0.8040)	No	2	36	3.91
B0X667	Trypsin 5	S (0.952)	17^18 (0.855)	inside: 291-293/Tmhelix: 268-290/outside: 1-267	65-NRTV (0.6702)/183-NVTV (0.8306)	151-T (0.653105)/159-S (0.523482)	3	13	3.59
B0XES8	Trypsin 7	S (0.832)	21^22 (0.665)	No	106-NVTF (0.6360)	No	3	36	3.11
B0X870	SP24D	S (0.891)	20^21 (0.801)	No	69-NGSV (0.6998)/75-NLSV (0.6183)	No	2	24	3.66
B0W9S9	Serine protease 1/2	S (0.960)	26^27 (0.750)	No	No	44-S (0.785129)	2	38	3.54
B0WW44	Cationic trypsin	S (0.926)	20^21 (0.794)	No	No	No	3	24	3.33

① TargetP 1.1 Server. Prediction of the subcellular location of trypsin. S = secreted. The number into the parenthesis indicates the probability

② SignalP 4.0 Server. Prediction of presence and location of signal peptide cleavage sites in the trypsin sequences. The numbers indicates the number of the amino acid residues involved in the cleavage. The number into the parenthesis indicates the probability

③ TMHMM 2.0 Server. Prediction of transmembrane helices in proteins. Tmhelix: transmembrane helix

④ NetNglyc 1.0 Server. Prediction of N-Glycosylation sites based on the presence of Asn-Xaa-Ser/Thr motifs. The number into the parenthesis indicates the probability

⑤ NetOglyc 4.0 Server. Prediction of mucin type GalNAc O-glycosylation sites

⑥ According to VectorBase database

Using VectorBase we analyzed the structure of the genes encoding the trypsin-like serine peptidases identified here. We observed that the exon number of the trypsin coding genes varies from one to three. With the exception of the Serine protease ½ that has an intron with 298 nucleotides, the other intron sequences are shorter than those observed in trypsin genes of vertebrates, varying between 25 and 71 nucleotides. Our analyses show that the intron exon structure is not conserved between all trypsin identified here, suggesting that several events of intron loss and gain have occurred in this species, which is in agreement with previous observations in other species [56, 62–64] (Table 3). In addition, the number of paralogues of each peptidase identified here was verified (Table 3). According to this analysis, the trypsin peptidases identified here have between 13 and 38 paralogues. The Trypsin1, 2, 4, 7, SP24D and Cationic trypsin are paralogues among them, suggesting that these peptidases were originated by gene duplication [14, 23, 65, 66]. In addition, the database mining shows that trypsin coding genes are generally clustered. For example, according to VectorBase, Trypsin1 is clustered with five other trypsin genes. It was suggested that the ancestors of dipterans had only one trypsinogen gene and that extra copies were gained by gene duplication [67]. In Culicidae, many trypsin-like serine peptidase coding genes are clustered in tandem arrays in different chromosomes, indicating that tandem duplication plays an important role in the expansion of this gene family [14, 20]. *C. quinquefasciatus* has the largest trypsin-like codifying gene repertoire when compared with other culicidae genomes [14, 23]. Such a peptidase repertoire may be associated with the ability of the insect to process blood components from different sources. In fact, this species has a high plasticity of feeding behavior, being able to feed on different species such as humans, dogs, birds and livestock [1–8]. Such a diversity of trypsin coding genes in this mosquito represents a substantial challenge for the assignment of putative functions, for determining their precise localization and mechanisms of regulation of expression. In fact, the understanding of the peptidase tissue expression patterns may be useful for the assignment of the putative function of such peptidases [64]. Thus, the use of techniques for the identification of active and tissue-specific peptidases in the midgut, as performed here, contributes for such function assignment.

The identification of active trypsin peptidases in *C. quinquefasciatus* females fed on sugar is in agreement with previous reports of our group that showed that *Ae. albopictus* females fed on sugar express active forms of trypsin [53]. The presence of active trypsin peptidases in sugar fed females of other mosquito species has been reported [20, 68, 69]. Several hematophagous diptera express a series of constitutive and blood meal-induced trypsin genes in the gut [16, 20, 22, 54]. The expression of peptidases in the midgut of sugar fed females may represent the induction of enzymes that was involved in the digestion of the larval/pupal meconium, or still induced by commensal bacteria into the midgut lumen [70]. In addition, because nondiapausing anautogenous mosquitoes need to feed on blood to complete the gonotrophic cycle, it is reasonable that they prepare their midgut tissue for blood digestion prior to blood feeding, so it is not surprising that after five days of adult emergence they express active trypsin peptidases. In fact, trypsin-coding genes were down regulated in anautogenous diapause-destined females. At the end of the diapause period (2–3 months at 18 °C), the expression of digestive peptidases increases, preparing the females for blood meal uptake [71]. Thus, the constitutive expression of trypsin peptidases could guarantee an efficient midgut metamorphosis and digestion of the blood meal, probably by zymogen activation, leading to improved biological fitness [55–57].

Despite molecular approaches that have allowed the identification of trypsin coding genes, the confirmation of the presence of these enzymes at protein level under different physiological conditions has not been reached, most likely because they are low abundance proteins [15, 24]. With the aim of analyzing whether we can develop selected reaction monitoring (SRM) experiments for detection of specific *C. quinquefasciatus* trypsin peptidases, we used the SKYLINE software for determining the occurrence of proteotypic peptides in those enzymes. SRM is a powerful method for monitoring target peptides within a complex protein sample and is particularly useful for hypothesis driven proteomics [72, 73]. Despite the presence of conserved motifs in the trypsin peptidases, the SKYLINE output shows that five out of the eight trypsin peptidases identified by mass spectrometry in our study have differences in amino acid sequences that allow the detection of unique peptides (Table 4). Remarkably, these unique peptides were the same identified by mass spectrometry in our study. The methodology used here for identifying proteotypic peptides can be used for developing SRM mass spectrometry assays for finding different trypsin peptidases in specific tissues or under specific stimulus. Noticeably, when we used the SKYLINE considering not only the sequences of the *C. quinquefasciatus* trypsin peptidases but also the sequences of those peptidases from other species with known genome sequences, such proteotypic peptides are both peptidase-specific and species-specific. This result is not conclusive because those genomes are not well annotated and in addition, the genomes of other related species have not yet been sequenced. However, the possibility to identified species-specific proteotypic peptides from trypsin peptidases is very interesting and should be followed.

**Table 4:** Proteotypic peptides from trypsins identified by MS/MS. Proteotypic peptides were predicted using SKYLINE software

Uniprot accession number	Protein name	Peptides identified by MS/MS	Species-specific peptide	Trypsin unique peptide	Other proteins witn the same peptide
B0WIS4	Trypsin 1	R.AAYVPAYNQNQCNSAYAR.Y	Yes	No	Q1KWX6 (Trypsin-like fragment) - C. quinquefasciatus
		R.IVGGFEISIADAPHQVSLQSR.G	Yes	Yes	-
		R.GSHICGGSIISPK.W	Yes	Yes	-
		R.NTIDYDYSLLELK.S	Yes	Yes	-
		K.WILTAAHCTDGASVSNLR.I	Yes	Yes	-
		K.HASGGSVISIK.R	-	-	Not predicted by Skyline
B0WE94	Trypsin 2	R.DEPADESQSLVSGWGDTR.S	No	No	Q962G7 / Q56GM3 (Trypsin) - Culex pipiens
		R.LEFGHAVQPVDLVR.D	No	No	Q962G7 / Q56GM3 (Trypsin) - Culex pipiens
		R.GVLVPLVNR.E	No	No	Q962G7 / Q56GM3 (Trypsin) - Culex pipiens
B0XCW2	Trypsin 4	K.GCAQPDYYGVYADVEK.A	Yes	Yes	-
		K.DFDFALLR.L	Yes	Yes	-
		R.VGSSYDYQGGTVIDVAGMTIHPR.Y	-	-	Not predicted by Skyline
B0X667	Trypsin 5	K.IIGGFPAEQGDTLHQVSIR.F	Yes	Yes	-
		K.GCGLAAYPGIYSDVAYYR.G	Yes	Yes	-
		R.GWIDSCLAGK.C	Yes	Yes	-
B0XES8	Trypsin 7	K.DACLGDSGGPLTCSGK.V	Yes	Yes	-
		R.DYALLNLAK.S	Yes	Yes	-
		R.AVDVPIADHDR.C	Yes	Yes	-
		R.GGQLIAVTR.K	-	-	Not predicted by Skyline
B0X870	SP24D	K.LGESIEYDELSQPIALYEGDDLPK.D	-	-	Not predicted by Skyline
B0W9S9	Serine protease 1/2	R.TGETFVDNQATVSGFGR.T	Yes	No	C. quinquefasciatus - Q23731 (Serine protease)
		R.TVDGGPVSPTK.N	Yes	No	C. quinquefasciatus - Q23731 (Serine protease)
B0WW44	Cationic trypsin	R.IVVHPQYAEGNLANDIAVIR.V	Yes	Yes	-

## Conclusion

The coupling of zymography, proteomic approaches and bioinformatic analyses, as performed here, shows to be a powerful approach in exploring the presence of active peptidases, which helps in the identification of genes that are in fact expressed at the protein level in a specific tissue. In this work, we identified eight different trypsin-like serine peptidases that have singularities at their gene organization level and at the protein sequence level. We identified and characterized trypsin peptidases that are expressed in the midgut of *C. quinquefasciatus*. The bioinformatics analysis conducted here allowed us to suggest that such trypsin peptidases could have primarily digestive functions. Importantly, we identified proteotypic peptide sequences that could be used in the future to directly identify trypsin peptidases in complex tissue-specific protein extracts of *C. quinquefasciatus*. This work represents the first step in the identification, at the protein level, of peptidases expressed in the *C. quinquefasciatus* midgut and in understanding their role in the complex physiological processes in such tissue.
